# Neoadjuvant Chemoradiotherapy Changes the Landscape of Soluble Immune Checkpoint Molecules in Patients With Locally Advanced Rectal Cancer

**DOI:** 10.3389/fonc.2022.756811

**Published:** 2022-04-21

**Authors:** Chao Liu, Peiliang Wang, Yi Sun, Xue Dou, Xiaoyu Hu, Wenxue Zou, Yanlai Sun, Qinyong Hu, Jinbo Yue

**Affiliations:** ^1^ Department of Oncology, Renmin Hospital of Wuhan University, Wuhan, China; ^2^ Department of Radiation Oncology, Shandong Cancer Hospital and Institute, Shandong First Medical University and Shandong Academy of Medical Sciences, Jinan, China; ^3^ Department of Radiation Oncology, Shandong Cancer Hospital and Institute, Cheeloo College of Medicine, Shandong University, Jinan, China; ^4^ Department of Surgical Oncology, Shandong Cancer Hospital and Institute, Shandong First Medical University and Shandong Academy of Medical Sciences, Jinan, China

**Keywords:** locally advanced rectal cancer, nCRT, soluble immune checkpoint molecules, sPD-L1, radiotherapy

## Abstract

**Background:**

We aimed to investigate clinical implications of specific soluble immune checkpoint molecules (sICMs) in locally advanced rectal cancer (LARC) patients treated with neoadjuvant chemoradiotherapy (nCRT).

**Methods:**

We prospectively enrolled 30 LARC patients treated with nCRT and collected blood samples from them before, during, and after nCRT for prospective studies. Immune checkpoints often refer to T cell surface molecules influencing the immune response. Immune checkpoints, in the form of a soluble monomeric form, is widely present in blood. In the study, eight immune checkpoint-related plasma proteins, including programmed death-ligand 1 (PD-L1), CD80, CD86, CD28, CD27, glucocorticoid-induced tumor necrosis factor receptor (GITR), GITR ligand (GITRL), and inducible T-cell costimulator (ICOS), were measured using the Luminex platform. Two independent pathologists categorized patients as the good responders and the poor responders according to Dworak tumor regression grade (TRG).

**Results:**

Of the 30 patients, the levels of sPD-L1, sCD80, sCD86, sCD28, sGITR, sGITRL, sCD27, and sICOS decreased during nCRT (Pre-nCRT vs. During-nCRT, all *p<*0.05) but were restored after nCRT treatment (Pre-nCRT vs. Post-nCRT, all *p>*0.05). In the 14 good responders, the levels of sICMs, other than sGITR (*p=*0.081) and sGITRL (*p=*0.071), decreased significantly during nCRT (Pre-nCRT vs. During-nCRT, *p<*0.05), but they were all significantly increased after nCRT (During-nCRT vs. Post-nCRT, all *p<*0.05). In the 16 poor responders, only sCD80 was significantly reduced during nCRT (Pre-nCRT vs. During-nCRT, p<0.05), and none was significantly increased after nCRT (During-nCRT vs. Post-nCRT, all p<0.05). High levels of sICMs before nCRT were associated with poor response (all OR≥1). The Pre-model that incorporated the 8 sICMs before nCRT yielded a good predictive value (AUC, 0.848) and was identified as an independent predictor of treatment response (OR, 2.62; 95% CI, 1.11-6.18; *p=*0.027).

**Conclusion:**

Our results suggest chemoradiotherapy could influence the change of sPD-L1, sCD80, sCD86, sCD28, sGITR, sGITRL, sCD27, and sICOS in patients with LARC. The levels of the majority of soluble immune checkpoint molecules were reduced during nCRT and then restored at the end of nCRT, particularly in patients who responded well to nCRT. Combined baseline sICMs can be developed to predict treatment response.

## Introduction

Colorectal cancer (CRC) is the third most common and the fourth leading cause of cancer-related deaths worldwide (1). Rectal cancer accounts for approximately 30% of CRC cases, with locally advanced rectal cancer (LARC) the most widespread form of rectal cancer (2). The standard treatment for LARC is neoadjuvant chemoradiation (nCRT), followed by total mesorectal excision. However, LARC’s sensitivity to nCRT varies among patients, with only 4%-20% of patients often achieving pathological complete response (pCR) after surgery (3).

Immune checkpoint inhibitors (ICIs), such as programmed cell death-1 inhibitor (PD-1) and cytotoxic T-lymphocyte antigen-4 (CTLA-4) inhibitor, have dramatically changed the treatment landscape of solid tumors recently. A high density of tumor-infiltrating lymphocytes (TILs), particularly CD8+ T lymphocytes, in rectal cancer is associated with better prognosis, suggesting that ICIs have promising antitumor effects in rectal cancer (4, 5).Radiation enhances CD8+ T cell infiltration in tumors and improves local tumor control, long-term survival, and protection against tumor rechallenge (6–8). The NICHE study (ClinicalTrials.gov: NCT03026140) showed that neoadjuvant treatment with ipilimumab plus nivolumab resulted in major pathological responses in 100% of mismatch repair (MMR)-deficient colon cancer cases without compromising surgery (9). There are ongoing clinical trials (NCT 03127007, NCT03102047, NCT02948348) evaluating the effectiveness of combining chemoradiation with ICIs to treat rectal cancer.

Reportedly, the soluble form of PD-L1 plays a role in immune suppression and is closely linked to poor prognosis in solids tumors (10–12). A study evaluating plasma soluble PD-1/PD-L1 levels in 117 advanced rectal cancer patients treated with nCRT revealed that high sPD-L1 levels after chemoradiation were associated with worse disease-free survival (13). However, no reports to date have examined the significance of the soluble form of co-stimulatory molecules, such as CD28-CD80/CD86, GITR-GITRL, CD27, and ICOS, in LARC patients treated with nCRT.

This study prospectively investigated the clinical implications of the changes in the soluble forms of PD-L1, CD80, CD86, CD28, GITR, GITRL, CD27, and ICOS in LARC patients treated with nCRT.

## Materials and Methods

### Study Population

LARC patients treated with nCRT between February 2018 and June 2019 were recruited prospectively. The eligibility inclusion criteria were as follows: (1) Histopathologically confirmed local advanced rectal adenocarcinoma without antitumor therapy; (2) pre-determined cT3-4N0-2M0 disease by pelvic magnetic resonance imaging (MRI); (3) Eastern Cooperative Oncology Group (ECOG) performance status of 0-2; (4) scheduled for nCRT and then surgery. Patients with autoimmune disease or having received immunosuppressive agents within 6 months before enrollment, were excluded. Clinicopathological features, including age, gender, tumor length, tumor location, lymphovascular invasion, capsular invasion, chemotherapy regimen, and RT plan were gathered and compared. The study was approved by the ethics committee of Shandong Cancer Hospital, and all patients gave written informed consent.

### Treatment

All patients received the standard treatment for locally advanced rectal cancer (neoadjuvant radiation with concurrent fluoropyrimidine-based chemotherapy, followed by surgical resection, including total mesorectal excision, **Table 1**). The patient’s response to nCRT were assessed histologically by two independent pathologists with expertise in gastrointestinal pathology. Dworak’s criteria was followed to evaluate tumor regression grade (TRG). Based on this grading, patients were divided into good responders (TRG 3/4) and poor responders (TRG1/2).

**Table 1:** Baseline demographic and clinical characteristics of patients.

Characteristic	TRG1-2 (n=16)	TRG3-4 (n=14)	Total (n=30)	P value
Age, median (range, yr)	59.5 (44.0-83.0)	53.5 (27.0-69.0)	55.5 (27.0-83.0)	0.085
Sex				0.257
Male	12 (75.0)	7 (50.0)	19 (63.3)	
Female	4 (25.0)	7 (50.0)	11 (36.7)	
Smoking status				0.709
Yes	6 (37.5)	4 (28.6)	10 (33.3)	
No	10 (62.5)	10 (71.4)	20 (66.7)	
Heavy alcohol use				0.689
Yes	5 (31.3)	3 (21.4)	8 (26.7)	
No	11 (68.8)	11 (78.5)	22 (73.3)	
BMI				0.715
≤23.7	7 (43.7)	8 (57.1)	15 (50.0)	
>23.7	9 (56.3)	6 (42.9)	15 (50.0)	
Cancer FHx				0.417
Yes	3 (18.7)	5 (35.7)	8 (26.7)	
No	13 (81.3)	9 (64.3)	22 (73.3)	
Polyps				1.000
Yes	6 (37.5)	5 (35.7)	11 (36.7)	
No	10 (62.5)	9 (64.3)	19 (63.3)	
Tumor length				1.000
<5 cm	5 (31.3)	4 (28.6)	9 (30.0)	
≥5 cm	11 (68.7)	10 (71.4)	21 (70.0)	
Tumor distance from anal verge				**0.014**
≤5cm	11 (68.7)	3 (21.4)	14 (46.7)	
>5cm	5 (31.3)	11 (78.6)	16 (53.3)	
pT stage				**0.024**
T0-2	3 (18.7)	9 (64.3)	12 (40.0)	
T3-4	13 (81.3)	5 (35.7)	18 (60.0)	
pN stage				0.260
N0	9 (56.3)	11 (78.6)	20 (66.7)	
N1-3	7 (43.7)	3 (21.4)	10 (33.3)	
Lymphovascular invasion				1.000
Yes	3 (18.7)	2 (14.3)	5 (16.7)	
No	13 (81.3)	12 (85.7)	25 (83.3)	
Perineural invasion				0.642
Yes	2 (12.5)	3 (21.4)	5 (16.7)	
No	14 (87.5)	11 (78.6)	25 (83.3)	
Induction chemotherapy				0.713
Yes	10 (62.5)	7 (50.0)	17 (56.7)	
No	6 (37.5)	7 (50.0)	13 (43.3)	
Chemotherapy regimen				1.000
CapeOX	14 (87.5)	13 (92.9)	27 (90.0)	
FOLFOX	2 (12.5)	1 (7.1)	3 (10.0)	
Radiotherapy plan				1.000
50 Gy/25 f	9 (56.3)	7 (50.5)	16 (53.3)	
50.4 Gy/28 f	7 (43.7)	7 (50.5)	14 (46.7)	

TRG, Dworak tumor regression grade; BMI, Body Mass Index; Cancer FHx, family history of cancer; CapeOX, capecitabine and oxaliplatin; FOLFOX, fluro-pyrimidine 5-FU, folinic acid, and oxaliplatin; Boldness indicates p-value less than 0.05.

### Plasma Samples and sICMs Measurements

Whole blood sample (10 mL) was collected from 30 patients before (1-3 days before nCRT), during (at the end of 14-15 fractional radiotherapy sessions), and after nCRT (1-2 days after nCRT completion). Blood samples were collected in tubes containing potassium EDTA (5 mL, Terumo Venogect II, Tokyo, Japan) and centrifuged at 1000 rpm at 4°C for 10 min within 30 min of collection to obtain plasma samples, then stored at -80°C.

The levels of plasma immune checkpoint protein biomarker profiles were determined quantitatively using the MILLIPLEX^®^ MAP Human Immuno Oncology Checkpoint Protein Panel (Cat. No. HCKPMAG-11K, Millipore Sigma). Samples were analyzed on a Luminex^®^ 200™ System and MILLIPLEX^®^ Analyst 5.1 software. All inter-assay and intra-assay coefficients of variation (CV) were below 15%.

### Statistical Analysis

The mean plasma immune checkpoint protein levels at each time were compared using the Wilcoxon signed-rank test. Correlations were analyzed according to the Gaussian distribution of data using Pearson’s chi-squared test. Weighted predictive score (wScore) models for tumor response were established from a linear combination of all sICMs and corresponding weights, which is implemented in the R package XGBoost. The predictive performance of each sICMs and model was quantified by the area under the ROC curve (AUC). Univariate and multivariate logistic regression analyses were conducted to determine independent prognostic factors of treatment response. Covariates identified by univariate analyses with a p value <0.10 were incorporated into the multivariate model constructed with the forward stepwise method. A two-sided p<0.05 was considered statistically significant. All statistical data were analyzed with R (http://www.r-project.org), SPSS 24.0, and the GraphPad 5.0 software.

## Results

### Characteristics of the Study Population

**Table 1** summarizes the clinical and pathological characteristics of the 30 patients. There were 19 men and 11 women, median age was 55.5 years (ranging from 27.0-83.0 years). They completed the standard neoadjuvant radiochemotherapy; specifically, the regimen included a radiotherapy session with 50.0 Gy in 25 fractions or 50.4 Gy in 28 fractions coordinated with CapeOX or FOLFOX chemotherapy. Patient demographics, including histology, previous chemotherapy, systemic treatment options, and number of metastases, were well-balanced between the two arms.

### sPD-L1, sCD80, sCD86, sCD28, sGITR, sGITRL, sCD27, and sICOS Levels Correlated Highly in LARC Patients

**Figure 1:**
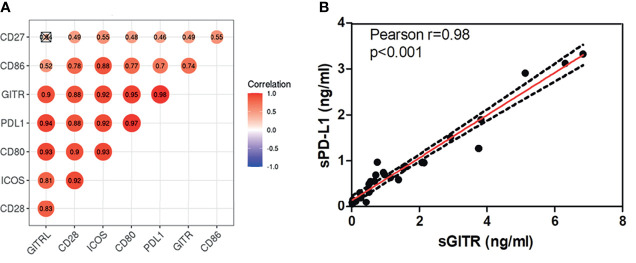
sPD-L1, sCD80, sCD86, sCD28, sGITR, sGITRL, sCD27, and sICOS levels correlated highly with each other in LARC patients. **(A)** Correlation between baseline plasma sPD-L1, sCD80, sCD86, sCD28, sGITR, sGITRL, sCD27, and sICOS in LARC patients. **(B)** Correlation between sGITR and sPD-L1 levels.

In this study, Immuno-Oncology Multiplex Assay Kits were utilized to measure levels of sPD-L1, sCD80, sCD86, sCD28, sGITR, sGITRL, sCD27, and sICOS. The 8 soluble immune checkpoint molecules were detected in all the patients. The median baseline levels were 0.62 ng/ml for sPD-L1 (ranging from 0.05 to 3.32 ng/ml), 0.55 ng/ml for sCD80 (ranging from 0.07 to 3.09 ng/ml), 5.01 ng/ml for sCD86 (ranging from 0.35 to 18.17 ng/ml), 16.51 ng/ml for sCD28 (ranging from 1.94 to 77.93 ng/ml), 0.74 ng/ml for sGITR (ranging from 0.01 to 6.84 ng/ml), 1.59 ng/ml for sGITRL (ranging from 0.19 to 9.69 ng/ml), 2.09 ng/ml for sCD27 (ranging from 0.35 to 4.56 ng/ml), and 7.00 ng/ml for sICOS (ranging from 0.77 to 26.10 ng/ml). **Figure 1A** shows a close positive correlation in the levels of soluble immune checkpoint molecules in patients, and there was only no statistical significance between sGITRL and sCD27 levels. The best correlation (r=0.94, *p<*0.001) occurred between sGITR and sPDL-1 levels (**Figure 1B**).

### Changes in sPD-L1, sCD80, sCD86, sCD28, sGITR, sGITRL, sCD27, and sICOS Levels Following nCRT

**Figure 2:**
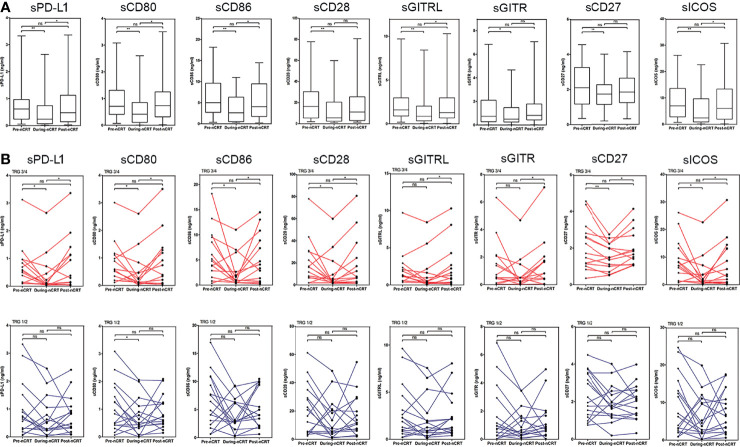
Changes in sPD-L1, sCD80, sCD86, sCD28, sGITR, sGITRL, sCD27, and sICOS levels following chemoradiotherapy. **(A)** Overall changes in all patients. **(B)** Individual changes in the good responders (TRG3/4 group) and the poor responders (TRG1/2 group).*p < 0.05, **p < 0.01, ns, not significant.

In all patients, the levels of sPD-L1, sCD80, sCD86, sCD28, sGITR, sGITRL, sCD27, and sICOS decreased during nCRT (Pre-nCRT vs. During-nCRT, all p<0.05), while were restored after nCRT treatment (Pre-nCRT vs. Post-nCRT, all p>0.05) (**Figure 2A**). In the 14 good responders, the levels of sICMs, other than sGITR (p=0.081) and sGITRL (p=0.071), decreased significantly during nCRT (Pre-nCRT vs. During-nCRT, p<0.05), but they were all significantly increased after nCRT (During-nCRT vs. Post-nCRT, all p<0.05) (**Figure 2B**). In the 16 poor responders, only sCD80 reduced significantly during nCRT (Pre-nCRT vs. During-nCRT, *p<*0.05), and none was significantly increased after nCRT (During-nCRT vs. Post-nCRT, all p<0.05) (**Figure 2B**).

**Figure 3:**
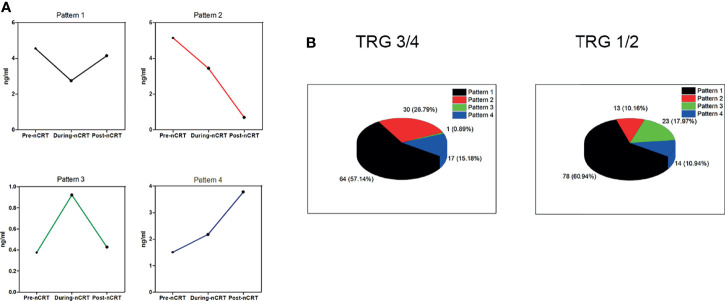
The change patterns of sPD-L1, sCD80, sCD86, sCD28, sGITR, sGITRL, sCD27, and sICOS. **(A)** Each soluble immune checkpoint molecule shows four different patterns of change based on three time-points. **(B)** The percentage of the four patterns in the TRG3/4 group (left) and the TRG1/2 group (right).

Our subsequent analysis of the pattern of sICMs’ variation shows that each immune checkpoint molecule changed in four different ways based on three time-points (**Figure 3A**). The change pattern was significantly different between the two groups. While patterns 1 and 4 accounted for similar proportions in both groups (the gap was less than 5%), the proportion of the pattern 2 in the good responder group was higher than that in the poor responder group (26.79% vs 10.16%), and the proportion of the pattern 3 in the good responder group was lower than that in the poor responder group (0.89% vs 17.97%) (**Figure 3B**).

### Plasma Levels of sPD-L1, sCD80, sCD86, sCD28, sGITR, sGITRL, sCD27, and sICOS Correlated Negatively With Tumor Response to nCRT

**Figure 4:**
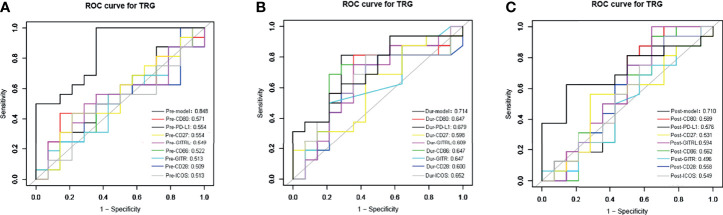
The receiver operating characteristic (ROC) curves analysis for the sensitivity and specificity of TRG classification. The ROC curve of sPD-L1, sCD80, sCD86, sCD28, sGITR, sGITRL, sCD27, sICOS, and the combined markers before **(A)**, during **(B)**, and after nCRT **(C)** were plotted. AUC (area under the curve) is in the left lower corner.

We used the ROC curve to analyze the predictive performance of each sICMs in treatment response at each time-point investigated. The ROC curves of each sICMs at each time-point show unsatisfactory predictive performances (AUC: 0.496-0.679) in **Figures 4A-C**. We then incorporated individual molecules to generate marker scores for better predictive performances at three time-points (**Supplementary Appendix**). The predictive performances of the three scores (AUC: 0.848 for Pre-Model, 0.714 for the During-Model, 0.710 for the Post-Model) were conformably superior to the predictive performances of the corresponding single molecules (**Figures 4A-C**).

**Figure 5:**
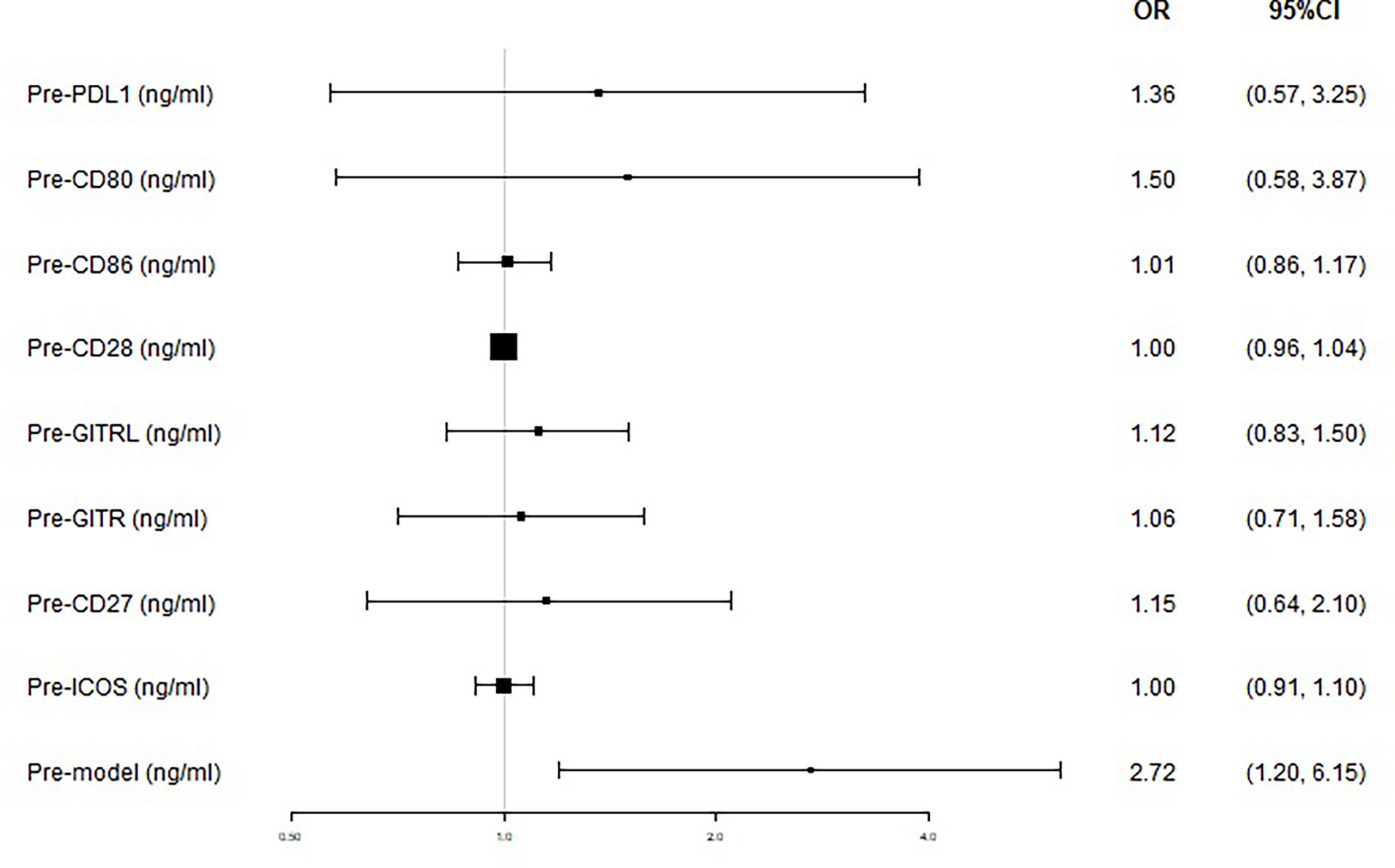
Treatment response according to baseline plasma levels of immune checkpoints. The prognostic relevance of each marker was assessed using the univariate logistic regression model and presented in the form of forest plot.

We next used logistic regression analyses to determine whether the levels of sICMs before nCRT could predict tumor response after nCRT. In the univariate analysis, high levels of sICMs, excluding sCD28 and sICOS, increased the risk of poor response but without statistical significance, while a high Pre-model score was significantly associated with poorer treatment response (OR, 2.72; 95% CI, 1.20 to 6.15; *p=*0.016) (**Figure 5**, **Table 2**). The multivariate model, after controlling for demographic and treatment variables, still revealed that the Pre-model was predictive of treatment response (OR, 2.62; 95% CI, 1.11-6.18; *p=*0.027) (**Table 2**).

**Table 2:** Univariate and multivariate analyses of factors associated with treatment response.

Characteristics	Univariate analysis			Multivariate analysis		
	OR	95% CI	p value	OR	95% CI	p value
Age (year)	1.06	0.99-1.14	0.095			NS
Gender (male vs female)	3.00	0.64-14.02	0.163	NI	NI	NI
Smoking (Yes vs No)	1.50	0.32-6.99	0.606	NI	NI	NI
Heavy alcohol use (Yes vs No)	1.67	0.32-8.74	0.546	NI	NI	NI
BMI (>23.7 vs ≤23.7)	1.71	0.40-7.29	0.466	NI	NI	NI
Cancer FHx (Yes vs No)	0.42	0.08-2.20	0.301	NI	NI	NI
Polyps (Yes vs No)	1.08	0.24-4.79	0.919	NI	NI	NI
Tumor distance from anal verge (<5 cm vs ≥5 cm)	1.14	0.24-5.46	0.873	NI	NI	NI
Tumor location (≤5 cm vs >5 cm)	0.60	0.11-3.15	0.546	NI	NI	NI
pT stage (T0-2 vs T3-4)	0.13	0.02-0.68	**0.016**	0.08	0.01-0.73	**0.026**
pN stage (N0 vs N1-3)	0.35	0.07-1.76	0.203	NI	NI	NI
Lymphovascular invasion (Yes vs No)	1.38	0.20-9.77	0.744	NI	NI	NI
Perineural invasion (Yes vs No)	0.52	0.07-3.70	0.517	NI	NI	NI
Induction chemotherapy (Yes vs No)	1.67	0.39-7.15	0.492	NI	NI	NI
Chemotherapy regimen (CapeOX vs FOLFOX)	0.54	0.04-6.67	0.630	NI	NI	NI
RT plan (50 Gy/25f vs 50.4 Gy/28 f)	1.29	0.31-5.43	0.732	NI	NI	NI
Pre-model (ng/ml)	2.72	1.20-6.15	**0.016**	2.62	1.11-6.18	**0.027**

BMI, Body Mass Index; Cancer FHx, family history of cancer; CapeOX, capecitabine and oxaliplatin; FOLFOX, fluro-pyrimidine 5-FU, folinic acid, and oxaliplatin; RT, Radiotherapy; CI, confidence interval; NI, not included in the multivariate model; NS, not statistically significant. Boldness indicates p-value less than 0.05.

### Association of Initial sPD-L1, sCD80, sCD86, sCD28, sGITR, sGITRL, sCD27, and sICOS Levels With Clinicopathological Characteristics

We divided the patients into a higher group and a lower group based on the median value of the each sICMs levels before nCRT. The relationships between clinicopathologic characteristics and sICMs levels were assessed using the Chi-square analysis. Only correlations between tumor location and sPD-L1, sCD80, sGITR, sGITRL were identified (all *p=*0.035, **Supplementary Table S1**). The correlation between the sICMs levels and other clinical variables, including age, sex, BMI, smoking status, heavy alcohol use, family history of cancer, polyps, tumor length, pT, pN, lymphovascular invasion, and perineural invasion were insignificant.

## Disussion

This study, to our knowledge, is the first prospective study to examined the significance of sPD-L1, sCD80, sCD86, sCD28, sGITR, sGITRL, sCD27, and sICOS in LARC patients treated with chemoradiotherapy. Our results showed that these immune checkpoint molecules correlate strongly with each other in LARC patients. This correlation may suggest a common source or regulation of these soluble proteins. The plasma levels of the 8 soluble immune checkpoint molecules decreaded during nCRT and were restored after nCRT, especially in responders to nCRT. The variation patterns differed significantly between the two groups, with more pattern 2 changes in good responders than in poor responders and more pattern 3 changes in poor responders than in good responders. Although single sICMs showed some prognostic relevance in the ROC curve and univariate logistic regression analyses, none of them was a good predictor of treatment response. To improve predictive performances, three scores incorporating these eight sICMs were constructed respectively at three time-points. The Pre-Model exhibited a higher predictive value than single sICMs and was identified as an independent predictor of treatment response.

In the immune system, immune checkpoints can be divided into co-inhibitory molecules, such as PD-1/PD-L1, and co-stimulatory molecules, such as CD80/CD86, ICOS/ICOS-L. The soluble form of immune checkpoint molecules is typically generated by the proteolytic cleavage of the membrane-bound form of the proteins, such as PD-L1 (14), or by the translation of alternatively spliced mRNA, such as CD80 (15) and CD86 (16).

The study described for the first time the changes of soluble immune checkpoint molecules (sPD-L1, sCD80, sCD86, sCD28, sGITR, sGITRL, sCD27, and sICOS) in patients with LARC following radiotherapy. The changes showed significant differences between responders and poor responders after nCRT. Good responders have more patients with decreased sICMs during CRT than poor responders; poor responders have more patients with increased sICMs during nCRT. These results suggest that chemoradiotherapy could remodel peripheral immune components. Additionally, one further points merit mention. Stereotactic body radiotherapy (SBRT) is more immunogenic than conventional fractionated radiotherapy (17). A recent study revealed that the two different radiotherapy fractioned regimens have a different effect on sPD-L1 changes; the sPD-L1 level continuously increased in the SBRT group but decreased after 1-month in the conventional fractionated radiotherapy group (18). However, the explanation for these phenomena is absent.

The sPD-1/sPD-L1 is the most widely studied soluble immune checkpoint molecule. Membrane-bound forms of PD-1 (mPD-L1) expressed on both tumor and immune cells, which may be the most prominent sources of sPD-L1 (14, 19). Preclinical studies have shown that radiotherapy increases the expression of PD-L1 in tumor lesions (6, 20). Hecht et al. examined PD-L1 expression in 63 pre-nCRT cases and matched them with post-nCRT sample expression to show that PD-L1 was upregulated in rectal cancer patients after nCRT (21). However, our results showed that sPD-L1 levels did not significantly increase after nCRT, which is inconsistent with its elevation after nCRT in published literature (13). Since our follow-up period was not long enough, further studies with a larger number of patients are required to clarify this contradictory point. In addition to changes of the sPD-L1 levels following radiotherapy, whether sPD-L1 can bind to PD-1 and deliver an inhibitory signal is debatable. Several studies have shown that elevated sPD-L1 is associated with poor prognosis in a wide variety of cancers, including malignant melanoma (22), lung cancer (23), hepatocellular carcinoma (24), multiple myeloma (25), and renal cell carcinoma (26). The study by Hyun Ju et al. firstly demonstrated that patients with a high level of initial sPD-L1 had a poor survival after RT in HCC patients (18). Similarly, our study found that patients with a high level of initial sPD-L1 trends to have poor response to chemoradiotherapy, although not statistically significant (**Figure 5**). These findings suggest that sPD-L1 might represent a biomarker for predicting the treatment response to chemoradiotherapy in LARC patients.

CD80/CD86-CD28 and ICOS-ICOSL belong to the B7-CD28 superfamily and provide important co-stimulatory signals to augment and sustain a T-cell response (27). GITR-GITRL and CD27 are important members of the TNF superfamily and also export co-stimulatory signals to support CD8 T-cell differentiation, proliferation, and NK cell functions (28). However, these soluble co-stimulatory molecules predominantly associate with poor prognosis in solid tumor patients (29–32), which is not consistent with the role of their corresponding membrane-bound forms in T cell activation. In our study, our result also support this phenomenon (**Figure 5**). One possible assumption for their association with poor prognosis is that their binding to corresponding immune checkpoint molecules in immune cells may prevent membrane receptor-ligand binding, thereby counteracting the co-stimulatory signals of T cells. Although the exact mechanism remains unclear, the soluble form of immune checkpoint molecules is demonstrated to involve in tumor immunity and affects the prognosis of patients.

However, none of the sICMs in our study was a reliable predictive marker. This, in fact, is not surprising. A single immune checkpoint molecule may not be powerful enough to predict treatment response. Multi-marker analyses that incorporate single markers into a panel may have more promising clinical application prospects. We also found that the Pre-model, incorporated baseline sICMs was an independent prognostic factor. Thus, there is promise for the development of a noninvasive blood-based model to serve as more convenient biomarker for the prediction of treatment response.

There are several limitations to our study. First, this study was a single-center exploratory analysis, and the sample size was limited. Second, chemotherapy might have influenced the levels of sICMs in our patients, although little is currently known about this possibility. Third, we did not validate our findings with another independent external study.

In conclusion, chemoradiotherapy significantly influenced the plasma levels of sPD-L1, sCD80, sCD86, sCD28, sCD27, and sICOS in LARC patients, and their changes differed markedly between good responders and poor responders. Patients with high baseline levels of these molecules tended to respond poorly to nCRT. A combined diagnostic panel that incorporates them had an obvious advantage over the corresponding single molecules. These findings suggest soluble immune checkpoint molecules have clinical values that may aid the future development of radiotherapy and immunotherapy.

## Data Availability Statement

The original contributions presented in the study are included in the article/**Supplementary Material**. Further inquiries can be directed to the corresponding authors.

## Ethics Statement

The studies involving human participants were reviewed and approved by the Research Ethics Board of Shandong Cancer Hospital. The patients/participants provided their written informed consent to participate in this study.

## Author Contributions

All authors contributed to the study conception and design. Data collection/preprocessing and analysis were performed by CL and PW. CL and PW contributed equally to the first draft of the manuscript, and all the listed authors revised the submitted manuscript and approved its final version before submission.

## Funding

This work was supported by the following grants: National Natural Science Foundation of China (Grant No. 81871895) and Young Taishan Scholars and Academic Promotion Program of Shandong First Medical University (Grant No. 2019RC003).

## Conflict of Interest

The authors declare that the research was conducted in the absence of any commercial or financial relationships that could be construed as a potential conflict of interest.

## Publisher’s Note

All claims expressed in this article are solely those of the authors and do not necessarily represent those of their affiliated organizations, or those of the publisher, the editors and the reviewers. Any product that may be evaluated in this article, or claim that may be made by its manufacturer, is not guaranteed or endorsed by the publisher.
