# Supplementary material for: Neoadjuvant Chemoradiotherapy Changes the Landscape of Soluble Immune Checkpoint Molecules in Patients With Locally Advanced Rectal Cancer
Source: Front Oncol. 2022 Apr 21;12:756811. doi: 10.3389/fonc.2022.756811 (PMC9070897; doi:10.3389/fonc.2022.756811)
Supplement: Supplementary file 1 [file DataSheet_1.docx]

Pre-model=-1.06593+12.70884*sPD-L1+11.83986*sCD80-0.23848*sCD86-0.02520*sCD28 -4.62792*sGITR -2.23057*sGITRL +0.09587*sCD27-0.73222*sICOS

During-model=-0.80896+5.69860*sPD-L1-0.29433*sCD80+0.38318*sCD86-0.10600*sCD28-1.76377* sGITR-0.33918* sGITRL+0.37188* sCD27 -0.03853* sICOS

During-model=-0.08438+2.60890*sPD-L1+3.31233*sCD80+0.15640*sCD86-0.07978*sCD28-0.52081*sGITR -0.07958*sGITRL -0.09727* sCD27 -0.39362* sICOS
